# Association of Travel Distance to Nearest Abortion Facility With Rates of Abortion

**DOI:** 10.1001/jamanetworkopen.2021.15530

**Published:** 2021-07-06

**Authors:** Kirsten M. J. Thompson, Hugh J. W. Sturrock, Diana Greene Foster, Ushma D. Upadhyay

**Affiliations:** 1 Bixby Center for Global Reproductive Health, Department of Obstetrics, Gynecology and Reproductive Sciences, University of California, San Francisco; 2 Department of Epidemiology and Biostatistics, University of California, San Francisco; 3 Advancing New Standards in Reproductive Health, Bixby Center for Global Reproductive Health, Department of Obstetrics, Gynecology and Reproductive Sciences, University of California, San Francisco

## Abstract

**Importance:**

Travel distance to abortion services varies widely in the US. Some evidence shows travel distance affects use of abortion care, but there is no national analysis of how abortion rate changes with travel distance.

**Objective:**

To examine the association between travel distance to the nearest abortion care facility and the abortion rate and to model the effect of reduced travel distance.

**Design, Setting, and Participants:**

This cross-sectional geographic analysis used 2015 data on abortions by county of residence from 1948 counties in 27 states. Abortion rates were modeled using a spatial Poisson model adjusted for age, race/ethnicity, marital status, educational attainment, household poverty, nativity, and state abortion policies. Abortion rates for 3107 counties in the 48 contiguous states that were home to 62.5 million female residents of reproductive age (15-44 years) and changes under travel distance scenarios, including integration into primary care (<30 miles) and availability of telemedicine care (<5 miles), were estimated. Data were collected from April 2018 to October 2019 and analyzed from December 2019 to July 2020.

**Exposures:**

Median travel distance by car to the nearest abortion facility.

**Main Outcomes and Measures:**

US county abortion rate per 1000 female residents of reproductive age.

**Results:**

Among the 1948 counties included in the analysis, greater travel distances were associated with lower abortion rates in a dose-response manner. Compared with a median travel distance of less than 5 miles (median rate, 21.1 [range, 1.2-63.6] per 1000 female residents of reproductive age), distances of 5 to 15 miles (median rate, 12.2 [range, 0.5-23.4] per 1000 female residents of reproductive age; adjusted coefficient, −0.05 [95% CI, −0.07 to −0.03]) and 120 miles or more (median rate, 3.9 [range, 0-12.9] per 1000 female residents of reproductive age; coefficient, −0.73 [95% CI, −0.80 to −0.65]) were associated with lower rates. In a model of 3107 counties with 62.5 million female residents of reproductive age, 696 760 abortions were estimated (mean rate, 11.1 [range, 1.0-45.5] per 1000 female residents of reproductive age). If abortion were integrated into primary care, an additional 18 190 abortions (mean rate, 11.4 [range, 1.1-45.5] per 1000 female residents of reproductive age) were estimated. If telemedicine were widely available, an additional 70 920 abortions were estimated (mean rate, 12.3 [range, 1.4-45.5] per 1000 female residents of reproductive age).

**Conclusions and Relevance:**

These findings suggest that greater travel distances to abortion services are associated with lower abortion rates. The results indicate which geographic areas have insufficient access to abortion care. Modeling suggests that integrating abortion into primary care or making medication abortion care available by telemedicine may decrease unmet need.

## Key Points

**Question:**

Is there an association between median travel distance to an abortion facility and abortion rate?

**Findings:**

In this cross-sectional geographic analysis of US counties, increases in median travel distance to the nearest abortion care facility were associated with significant reductions in median abortion rate (21.1 per 1000 female residents of reproductive age for <5 miles; 3.9 per 1000 female residents of reproductive age for ≥120 miles). Reductions in travel distances were associated with significant increases in the median abortion rate (telemedicine simulation, 10.2 per 1000 female residents of reproductive age).

**Meaning:**

In this study, the abortion rates declined as travel distance to an abortion care facility increased, and modeling suggests the need for abortion care can be only partially met through service delivery innovations.

## Teaser

This cross-sectional geographic study examines the association between travel distance to the nearest abortion care facility and abortion rate and models the effect of reduced travel distance among US female residents of reproduction age.

## Introduction

In the US, increasing travel distance or travel time to a health care clinician is associated with less use of preventive care and poorer health outcomes for women, including reduced use of mammography,^1,2^ later stage at diagnosis of breast cancer,^3,4^ and reduced use of risk-appropriate colonoscopy.^5^ County-level analyses of pregnancy-related outcomes have shown spatial relationships in rates of prenatal care use,^6^ and the closure of rural hospitals not adjacent to urban areas was associated with increased preterm births in the following year.^7^

Abortion is a common reproductive health care service, with 1 in 4 US women obtaining this care during their lifetime.^8^ However, many states have implemented policies restricting abortion care clinicians and facilities (hereinafter referred to as abortion providers).^9^ Studies of these policies have documented clinic closures and women unable to obtain abortion care, with disproportionate effects on low-income women and non-White women.^10,11^ Increased travel for an abortion is associated with delays in care, increased costs, and stress.^10,12^ Even when women are able to obtain abortion care, greater travel distance has been associated with decreased odds of returning to the abortion facility for follow-up care and increased odds of visiting an emergency department.^13^

Research in a variety of settings has indicated that the farther a woman lives from an abortion care facility, the less likely she is to obtain that care. These studies used distance or travel time to an abortion provider as a measure of potential rather than realized access.^14^ Regional research has focused on California, Texas, New York, and Wisconsin^11,15,16,17,18^; national analyses have focused on disparities in access.^19,20,21^ One longitudinal, econometric study in 18 states^22^ found an association between travel distance and abortion rate but did not generate interpretable abortion rates.

We conducted a national analysis to test the hypothesis that greater travel distance to the nearest abortion facility is associated with lower abortion rates and to provide estimated abortion rates under actual conditions and alternate assumptions of abortion access. We extend the literature by estimating changes in abortion rate under 2 travel distance scenarios: less than 30 miles (48 km), a common definition of network adequacy for primary care,^23^ and less than 5 miles (8 km), a simulation of medication abortion by telemedicine.

## Methods

### Study Design

This geographic analysis used publicly available data from 27 states, the American Community Survey, and the US Census to calculate county-level abortion rates per 1000 female residents of reproductive age (15-44 years) in 2015, the most recent year of county-level data available when the study began. We compare abortion rates across counties with varying median travel distances to the nearest abortion facility. We estimated the abortion rate for 48 states and estimated the effect of different travel distance scenarios on the abortion rate in a multivariable model. Data were collected from April 2018 to October 2019. The University of California, San Francisco, institutional review board approved this study and waived the need for informed consent for the use of publicly available data. This study followed the Strengthening the Reporting of Observational Studies in Epidemiology (STROBE) reporting guideline.

### Study Population

A county or an equivalent administrative unit is the smallest geographic unit for which abortions are reported and was the unit of analysis for this study. We limited our analyses to the 48 contiguous US states (n = 3107 counties) because modes of travel other than by road are common in Hawaii and Alaska. To develop the model for estimations, we used data from 27 states that publicly report abortion by county of residence (n = 1948 counties). When states’ reports disagreed with the Centers for Disease Control and Prevention abortion estimates by state of residence for 2015 by greater than 1%, we adjusted the number of abortions.^24^ Adjustments increased the abortion count by 4.8% overall, with the largest changes in states reporting fewer than 10 000 abortions. Seven states suppressed abortion counts for counties reporting fewer than a specific number of abortions (eg, <10). In these 267 counties, we replaced the suppressed count with the midpoint between 0 and the specified limit (eg, 5) when it did not create a county abortion rate exceeding the state mean; otherwise, the county abortion count was missing. To calculate county-level abortion rate per 1000 female residents aged 15 to 44 years, we used 2015 American Community Survey 5-year population estimates.

### Exposures and Outcomes

The study outcomes were numbers of abortions and abortion rate per 1000 female residents of reproductive age by county of residence. The main exposure was the travel distance to the nearest abortion facility. We used the Advancing New Standards in Reproductive Health national abortion facility database and Open Source Routing Machine to determine travel distance and time by car.^20,25^ We included publicly advertised abortion facilities operating in 2015, as verified by telephone calls, local news reports, or a website active throughout the year. We identified latitude/longitude coordinates for each abortion facility (n = 789), calculated travel distance from the population-weighted center of each census tract (n = 72 539), selected the facility with the shortest travel distance, and calculated the median travel distance per county.^26^ We excluded census tracts with no residents and those with only institutionalized male populations. We used a categorical variable for travel distance because previous research has shown a nonlinear relationship between travel distance and abortion rates.^11,15,16,17,18^ To determine the cut points for the categorical variable, we used state legislatures’ common definitions of health care network adequacy.^23^

For scenario testing, the main exposures were travel distance of less than 30 miles, a common definition of network adequacy for primary care, or less than 5 miles, simulating medication abortion by telemedicine. We considered the shortest travel distance in our model to simulate medication abortion by telemedicine because patients still spend time seeking and obtaining these services.

### Control Variables

Analyses included covariables identified as associated with the abortion rate: age, race/ethnicity, marital status, educational attainment, household income, and nativity.^1^ Race/ethnicity data were based on self-report in the US Census. Other covariables were from the American Community Survey 5-year estimates for 2015. We used a 5-level state policy grade (A, B, C, D, or F) developed by the National Abortion Rights Action League for 2015 that captures “the cumulative burden each state imposes on access to reproductive health care.”^27^^(p78)^ The grade has been used in previous studies on abortion policies^28^ and takes into account enacted laws and regulatory activity, such as whether advanced practice clinicians may provide early abortion care, whether state Medicaid covers abortion care, whether waiting periods or in-person counseling are mandated, and whether laws target abortion care facilities or ban some types of abortion.

### Statistical Analysis

Data were analyzed from December 2019 to July 2020. To model abortion rates, we used a spatial Poisson generalized additive model. We modeled rates using numbers of abortions as the outcome and population of female residents of reproductive age as an offset term. We included a priori covariables as linear terms (county proportions of female residents aged 25-29 years; non-Hispanic Black, Asian, Native American, Alaska Native, Native Hawaiian, Pacific Islander, or multiracial female residents aged 15-44 years; married women older than 18 years; women aged 18-44 years with a high school degree or equivalent; households with female residents aged 15-44 years below the federal poverty level; and foreign-born female residents), except travel distance and state policy grade, which were categorical. To account for residual spatial autocorrelation, we fit a spatial smooth across counties using a Markov random field (MRF).^29^ To tune the parameters of the MRF, including the snap parameter defining the neighborhood matrix and the smoothing parameter, we used cross-validation. We created 27 cross-validation folds using counties grouped by state, each with a validation set of counties belonging to the same state. We took this approach because missing county data were mostly the result of entire states not reporting. We compared models using cross-validated mean squared error and residual spatial autocorrelation (Moran I statistic) and by examining estimated rates to identify models that fell outside a plausible maximum (>100 per 1000 female residents of reproductive age).

To make estimations, including the travel distance scenarios, we first established which counties lay outside the spatial limits of the observed data. For those counties outside the convex hull of observed data, we made estimations based on the covariables alone. This process was based on experiments that showed that extrapolating the spatial effect can lead to implausible estimated rates. For counties within the convex hull of the observed data, the estimations use the full spatial model. As a sensitivity analysis, we used a categorical variable for travel time as the main exposure.

We used R, version 3.5.3, and the mgcv package for modeling and estimation, version 1.8-27 (R Foundation for Statistical Computing). Statistical tests were 2 sided and used *P* < .05 to indicate statistical significance.

## Results

Counties that reported (n = 1948) vs those that did not report (n = 1160) residents’ abortions had similar mean numbers of female residents aged 25 to 29 years (15.6% vs 15.6%), high school–educated women aged 18 to 44 years (27.2% vs 27.8%), foreign-born female residents (4.5% vs 4.4%), and female residents of reproductive age living in households below the federal poverty level (22.1% vs 22.1%) (Table 1). Meaningful differences between states that report vs those that do not report county-level abortions were the proportion of Black residents of reproductive age (10.4% vs 6.1%) or those of other race/ethnicity (3.4% vs 5.5%), the proportion of married female residents older than 15 years (50.7% vs 51.7%), and the distribution of reproductive health policy grades (eg, grade F, 41.7% vs 15.2%).

**Table 1.** Selected Sociodemographic Characteristics of Study Population by Whether Counties Report Residents’ Abortions (2015)

Characteristic	No. of female residents (mean %)	
	Counties reporting abortions (n = 1948)	Counties not reporting abortions (n = 1160)
Women aged 25-29 y	6 321 800 (15.6)	4 309 660 (15.6)
Race/ethnicity		
Black	5 527 770 (10.4)	3 056 010 (6.1)
Other^a^	2 619 280 (3.4)	2 730 010 (5.5)
Married	37 727 100 (50.7)	25 897 450 (51.7)
High school or equivalent degree	7 530 720 (27.2)	5 151 650 (27.8)
Foreign born	10 104 930 (4.5)	11 096 240 (4.4)
Household below federal poverty level	7 251 190 (22.1)	4 809 360 (22.1)
State policy grade^b^		
A	2 161 040 (3.4)	8 887 200 (14.2)
B	6 664 510 (10.6)	4 226 790 (6.7)
C	2 301 990 (3.7)	2 195 690 (3.5)
D	NA	447 280 (0.7)
F	26 149 050 (41.7)	9 505 470 (15.2)

Abbreviation: NA, not applicable.

^a^ Includes Asian, Native American, Alaska Native, Native Hawaiian, Pacific Islander, and multiracial residents.

^b^ Only 2 states (West Virginia and Wyoming) have a D grade, and neither report abortions by county of residence. Washington, DC, is missing a policy grade.

**Figure 1.**
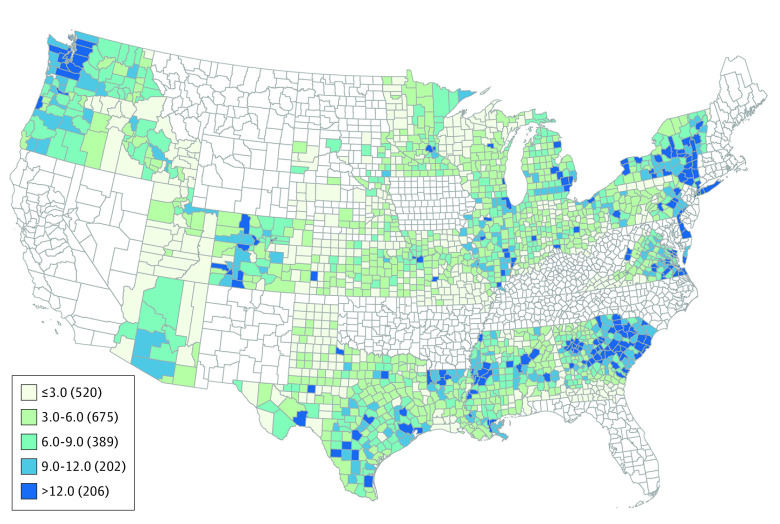
Reported Abortion Rate per 1000 Women of Reproductive Age, by County of Residence (2015) Includes female residents aged 15 to 44 years.

In 27 states and 1948 counties with 37.3 million female residents of reproductive age, there were 428 720 reported abortions (eTable 1 in the Supplement and Figure 1). The mean abortion rate was 11.5 per 1000 female residents of reproductive age; the median rate was 9.9 per 1000 female residents of reproductive age (range, 0-63.6 per 1000 female residents of reproductive age). The Moran I statistic showed a strong spatial correlation of reported abortions, at 0.49. In 48 states, the population-weighted mean travel distance to the nearest facility offering abortion care was 25.6 miles (41 km); median travel distance, 8.2 miles (13 km) (range, 1-383 miles [1.6-613 km]) (eFigure 1 in the Supplement).

In a multivariable spatial Poisson model, greater travel distance was associated with declining abortion rates in a dose-response association (Table 2). Compared with a median county travel distance of less than 5 miles, the abortion rate declined by 0.05 (95% CI, −0.07 to −0.03) per 1000 female residents of reproductive age at 5 to less than 15 miles (8 to <24 km), 0.22 (95% CI, −0.24 to −0.19) per 1000 female residents of reproductive age at 15 to less than 30 miles (24 to <48 km), 0.34 (95% CI, −0.37 to −0.31) per 1000 female residents of reproductive age at 30 to less than 60 miles (48 to <96 km), 0.43 (95% CI −0.47 to −0.39) per 1000 female residents of reproductive age at 60 to less than 120 miles (96 to <193 km), and 0.73 (95% CI −0.80 to −0.65) per 1000 female residents of reproductive age at 120 miles or more (≥193 km). The cross-validated mean squared error for this model was 0.01, and the Moran I statistic was −0.01.

**Table 2.** Decline in County-Level Abortion Rate in a Spatial Poisson Model, by Travel Distance to the Nearest Abortion Care Facility (n = 1948)

Travel distance, miles^a^	No. of counties	No. of female residents of reproductive age^b^	No. of abortions	Median abortion rate (range)^c^	Coefficient (95% CI)	
					Unadjusted	Adjusted^d^
<5	62	7 949 470	167 520	21.1 (1.2-63.6)	1 [Reference]	1 [Reference]
5 to <15	157	13 358 870	160 710	12.2 (0.5-23.4)	−0.31 (−0.33 to −0.29)	−0.05 (−0.07 to −0.03)
15 to <30	179	4 902 670	37 850	7.5 (0.7-25.2)	−0.77 (−0.79 to −0.75)	−0.22 (−0.24 to −0.19)
30 to <60	520	4 836 870	30 680	5.9 (0.2-19.3)	−0.80 (−0.82 to −0.78)	−0.34 (−0.37 to −0.31)
60 to <120	709	4 905 300	26 660	5.1 (0.0-17.6)	−0.85 (−0.88 to −0.81)	−0.43 (−0.47 to −0.39)
≥120	321	1 323 420	5310	3.9 (0.0-12.9)	−1.13 (−1.20 to −1.06)	−0.73 (−0.80 to −0.65)

^a^ To convert to kilometers, multiply by 1.6.

^b^ Includes women and girls aged 15 to 44 years.

^c^ Indicates population-weighted travel distance category median per 1000 women aged 15 to 44 years.

^d^ Adjusted for county-level proportion of women aged 25 to 29 years, Black or other race/ethnicity, married, high school degree or equivalent, foreign-born residents, households below the federal poverty level, and state abortion policies.

**Figure 2.**
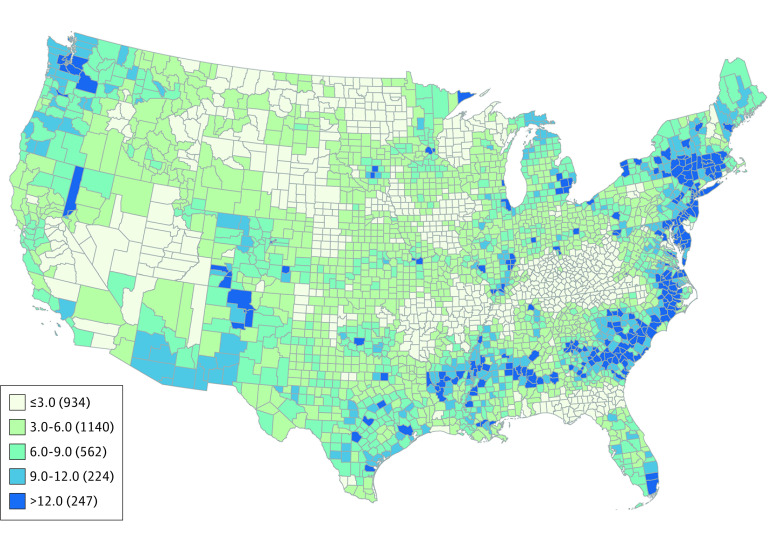
Estimated Abortion Rate per 1000 Women of Reproductive Age, by County of Residence (2015) Includes female residents aged 15 to 44 years.

Based on this model, we estimated the abortion rate in the 48 contiguous states using 2015 travel distance conditions. The national estimate is for 3107 counties with 62.5 million female residents of reproductive age. We estimated 696 760 abortions with a mean abortion rate of 11.1 and median abortion rate of 8.7 per 1000 female residents of reproductive age (range, 1.0-45.5) (Table 3 and Figure 2).

**Table 3.** Estimated Increases in Abortions and Abortion Rate Under Travel Distance Scenarios, by State (2015)^a^

State	No. of counties	No. of female residents of reproductive age^b^	Actual travel distance		Travel distance scenario <30 miles^c^		Travel distance scenario <5 miles^c^	
			No. of abortions	Median rate (range)^d^	No. of abortions	Median rate (range)^d^	No. of abortions	Median rate (range)^d^
All	3107	62 539 010	696 470	8.7 (0.9-45.5)	714 660	9.3 (1.1-45.5)	767 390	10.2 (1.4-45.5)
Alabama	67	961 050	6730	5.6 (1.8-19.4)	7090	5.6 (2.3-22.0)	7990	6.3 (2.8-27.4)
Arizona	15	1 296 680	12 620	11.5 (2.1-11.5)	13 380	11.5 (3.4-12.4)	14 820	12.1 (4.3-15.5)
Arkansas	75	575 660	2260	2.9 (1.0-9.5)	2530	3.1 (1.6-12.2)	2970	3.9 (1.9-15.3)
California	58	8 017 350	57 860	7.5 (1.2-10.2)	57 980	7.5 (1.4-10.2)	59 080	7.5 (1.8-10.7)
Colorado	64	1 072 350	8970	8.1 (2.0-12.7)	9100	8.1 (3.3-14.6)	9640	8.5 (4.2-18.2)
Connecticut	8	686 250	18 230	12.3 (6.2-90.0)	18 230	12.3 (6.2-90.0)	18 700	12.3 (6.6-94.9)
Delaware	3	179 990	2970	18.2 (13.2-18.2)	3030	18.2 (14.0-18.2)	3170	18.2 (14.8-18.6)
Florida	67	3 661 230	38 290	9.4 (0.3-21.0)	38 530	9.4 (0.4-21.0)	40 130	9.7 (0.4-21.0)
Georgia	159	2 099 370	26 740	12.3 (0.6-25.3)	27 650	14.5 (0.7-25.3)	30 820	15.9 (0.8-25.3)
Idaho	44	311 320	1680	5.6 (1.1-7.8)	1800	5.7 (1.4-10.3)	2010	6.6 (1.7-12.8)
Illinois	102	2 604 060	35 430	10.6 (2.6-18.3)	36 120	10.6 (2.9-25.3)	38 220	12.2 (3.7-31.5)
Indiana	92	1 291 070	9590	6.2 (1.5-16.2)	10 180	6.8 (1.5-16.2)	11 400	8.2 (1.7-16.2)
Iowa	99	584 640	1980	3.4 (0.3-6.6)	2100	3.7 (0.3-7.9)	2370	4.5 (0.3-9.8)
Kansas	105	558 930	3670	6.8 (1.1-9.6)	4100	7.2 (1.9-11.9)	4710	8.4 (2.4-14.8)
Kentucky	120	854 350	2230	2.2 (0.5-9.3)	2490	2.2 (0.6-9.5)	2850	2.3 (0.7-11.9)
Louisiana	64	939 820	8510	8.4 (1.6-18.9)	9300	10.1 (2.7-19.1)	10 530	11.5 (3.3-23.8)
Maine	16	236 080	2370	9.6 (5.4-12.6)	2500	10.4 (8.3-12.6)	2880	12.0 (10.4-13.3)
Maryland	24	1 200 200	24 420	19.5 (0.1-50.3)	24 510	19.5 (0.1-50.3)	25 220	19.5 (0.1-54.7)
Massachusetts	14	1 366 220	26 530	13.2 (4.9-68.5)	26 590	13.2 (6.1-68.5)	28 610	14.0 (7.6-72.2)
Michigan	83	1 892 790	26 280	12.1 (0.2-29.1)	26 920	12.1 (0.3-29.1)	28 530	13.3 (0.4-29.1)
Minnesota	87	1 049 650	9150	7.6 (0.6-15.9)	9600	8.2 (0.8-15.9)	10 920	10.2 (1.0-16.8)
Mississippi	82	601 390	4720	7.1 (2.5-18.2)	5380	7.9 (3.1-22.4)	6430	9.9 (3.9-27.9)
Missouri	115	1 175 400	8530	6.2 (1.0-13.9)	9000	6.4 (1.6-13.9)	10 010	7.7 (2.0-14.7)
Montana	56	183 610	760	3.9 (1.6-8.9)	860	4.4 (2.1-10.0)	980	5.1 (2.6-12.4)
Nebraska	93	362 370	1930	5.5 (0.6-7.9)	2070	5.9 (1.0-7.9)	2290	6.4 (1.2-8.8)
Nevada	17	560 790	5170	8.5 (1.1-15.1)	5220	8.5 (1.8-15.1)	5570	9.0 (2.3-18.6)
New Hampshire	10	244 820	1540	6.4 (1.1-10.3)	1550	6.4 (1.4-10.3)	1820	6.7 (1.8-10.9)
New Jersey	21	1 726 130	43 320	18.4 (0.0-87.3)	43 330	18.4 (0.0-87.3)	44 630	18.4 (0.1-92.0)
New Mexico	33	398 840	4100	9.8 (2.6-29.6)	4490	12.1 (4.4-33.6)	5060	13.5 (5.5-41.8)
New York	62	4 048 540	88 620	17.2 (4.9-45.5)	88 770	17.2 (4.9-45.5)	90 170	17.7 (5.2-45.5)
North Carolina	100	1 974 620	17 860	6.7 (0.8-28.1)	18 870	6.7 (1.0-31.2)	21 730	8.0 (1.3-38.9)
North Dakota	53	139 430	260	1.7 (0.9-4.3)	360	2.1 (1.0-7.2)	440	2.7 (1.3-8.9)
Ohio	88	2 214 450	21 220	8.3 (1.7-20.9)	21 730	8.3 (2.1-20.9)	24 060	10.0 (2.6-20.9)
Oklahoma	77	755 550	4270	5.7 (1.3-10.4)	4610	5.7 (1.5-12.9)	5240	7.0 (1.8-16.0)
Oregon	36	770 670	7750	10.0 (1.6-14.1)	7910	10.0 (2.7-14.1)	8500	10.7 (3.3-14.3)
Pennsylvania	67	2 418 790	32 060	9.6 (2.2-35.2)	32 800	10.4 (2.8-35.2)	35 080	11.5 (3.5-35.2)
Rhode Island	5	210 990	5230	35.9 (3.4-35.9)	5230	35.9 (3.4-35.9)	5550	37.9 (4.2-37.9)
South Carolina	46	937 780	10 960	11.3 (5.4-21.9)	11 720	12.2 (6.1-27.1)	13 850	14.7 (7.6-33.7)
South Dakota	66	156 850	710	3.9 (0.5-13.3)	840	4.9 (0.8-16.4)	980	6.1 (1.0-20.5)
Tennessee	95	1 290 200	8330	5.3 (0.5-13.4)	8690	6.0 (0.7-13.4)	9880	6.6 (0.8-14.1)
Texas	254	5 577 580	54 220	9.5 (1.8-13.4)	57 620	9.6 (2.8-13.5)	65 210	11.9 (3.4-15.7)
Utah	29	631 170	3150	4.1 (0.5-7.9)	3340	4.6 (0.9-8.4)	3800	5.7 (1.1-10.4)
Vermont	14	116 670	1150	9.6 (2.3-17.4)	1170	9.6 (2.3-17.4)	1320	10.2 (2.9-18.3)
Virginia	133	1 677 520	18 490	9.2 (0.5-35.8)	18 920	10.2 (0.5-35.8)	20 520	11.1 (0.7-40.6)
Washington	39	1 390 380	17 320	13.0 (2.4-14.8)	17 510	13.0 (3.0-14.8)	18 310	13.7 (3.7-17.9)
West Virginia	55	337 720	1200	2.9 (0.6-10.5)	1410	3.3 (0.8-13.0)	1740	3.7 (1.0-16.1)
Wisconsin	72	1 088 170	6740	4.9 (0.2-13.6)	7070	5.2 (0.2-13.6)	8050	6.5 (0.3-14.4)
Wyoming	23	109 560	330	2.5 (0.8-5.8)	460	3.7 (1.4-7.2)	570	4.6 (1.7-8.9)

^a^ Columns may not sum to total due to rounding.

^b^ Indicates women and girls aged 15 to 44 years.

^c^ To convert to kilometers, multiply by 1.6.

^d^ Rates are population-weighted median per 1000 female residents aged 15 to 44 years.

In estimates under 2 alternate travel scenarios, lesser travel distance was associated with changes in the abortion rate (Table 3 and eTable 2 in the Supplement). With all travel distances less than 30 miles, a common definition of network adequacy for primary care,^23^ we estimated 714 660 abortions, a 2.6% increase (18 190 additional abortions) (eFigure 2 in the Supplement). In this scenario, the mean abortion rate was 11.4 per 1000 female residents of reproductive age; the median rate was 9.3 per 1000 female residents of reproductive age (range, 1.1-45.5). In the scenario with all travel distances of less than 5 miles, a simulation of medication abortion by telemedicine, we estimated 767 390 abortions, a 10.2% increase (70 920 additional abortions) (eFigure 3 in the Supplement). In this scenario, the mean abortion rate was 12.3 per 1000 female residents of reproductive age and the median rate was 10.2 per 1000 female residents of reproductive age (range, 1.4-45.5). Under both travel distance scenarios, the largest increases in abortion rate were in South Carolina, Mississippi, New Mexico, Louisiana, Wyoming, Texas, North Carolina, Georgia, South Dakota, and Arizona. States with the smallest estimated increases in abortion rate were California, New York, Colorado, Iowa, Connecticut, Maryland, Nevada, and Washington. In sensitivity analyses using travel time as the main exposure, the results were similar (eTable 3 in the Supplement).

## Discussion

This study provides a national model of abortions by county of residence and confirms the association between greater travel distance and lower abortion rates previously found in state-level and regional analyses.^11,15,16,17,18,19,20,21,22^ The declines in the median abortion rate by travel distance category are clinically meaningful and have meaningful consequences for people’s lives. This study generated interpretable state- and county-level abortion rates that have utility for policy makers. It also estimates the unmet need for abortion services associated with large travel distances from facilities. As states continue to enact laws restricting abortion access, it is likely that the distance to care will grow for more US women. Implementing policies to reduce spatial inequalities will become even more important in the event that the US Supreme Court returns to states the right to ban abortion. Even with *Roe v Wade* intact, we estimate there would be more than 70 000 additional abortions annually if spatial inequalities were eliminated.

Our findings regarding the association between travel distance and abortion rate are consistent with previous regional studies, suggesting this phenomenon is robust to differing analytic approaches.^11,15,16,17,18^ Most regional analyses used differences-in-differences to estimate the decrease in abortion rate after state policy changes and abortion facility closures. Unlike Brown et al,^22^ we did not find evidence of a threshold association between distance to abortion provider and the abortion rate, possibly because our distance measure was more precise, based on road travel to the address of the nearest facility rather than the straight-line distance between population-weighted centers of 2 counties. This analysis expands what is known about the association between travel distance and abortion rates by estimating the effect of public health interventions that could reduce travel for abortion care. We estimate the number of individuals who would want abortion care but are unable to access it due to long travel distances.

One critique of this analysis is that reverse causality may be at play—that is, facilities providing abortion care may locate where there is demand, making the observed association between abortion rate and travel distance a function of declining demand. In a study designed to address reverse causality, Brown et al^22^ showed that increased distance to abortion providers accounted for their observed declines in the abortion rate. This is plausible because state laws and regulations of abortion providers directly affect where they can locate, constraining a demand-driven response.^30^ To address this critique, we account for state abortion policies by including a covariable in our model. When spatial inequalities are eliminated in the telemedicine scenario, all but 1 of the states with the greatest increases in abortion rate have highly restrictive abortion policies (grade D or F): New Mexico (grade B) had a large increase in abortion rate and supportive policies, likely due to large travel distances to an abortion provider (73% of counties have median travel distance >60 miles). Conversely, states with the smallest increases in abortion rate all have policies supportive of access to abortion care and low numbers of women of reproductive age per facility.

A periodic census of US abortion providers suggests a larger number of abortions than our model estimates, although the census does not provide data for 2015.^1^ Our model’s underestimation of abortions in 2 populous states at the edges of the map, Florida and California, accounts for most of the difference between these 2 sources of data. Our model underestimates abortion in these states because neither California nor Florida report abortions by county of residence and because the MRF smooth term trends toward zero along adjacency map edges. We were able to partially address this by using the MRF smooth term only in counties that fall within the convex hull of reported data. This underestimation means that our estimates of the number of abortions in all scenarios are conservative.

### Limitations

Some limitations of this study are shared by all abortion-related research. Abortions are underreported by states for a variety of reasons, so our model likely underestimates the abortion rate. Our model includes more states with more complete data than previous studies and had fewer differences in female sociodemographic characteristics by counties’ abortion reporting status. Travel distance to an abortion facility is not the only barrier faced by people seeking abortion care; financial limitations, restrictive laws, religious beliefs, and stigma also create barriers.^31^ The model partly addresses these barriers by including household income and reproductive health state policy grades as covariables.^27^

Some limitations of this study are specific to spatial analyses. Outcomes are sometimes sensitive to the level of geography used in an analysis.^32^ We had no option but to use county as the geographic unit of analysis owing to the way abortion data are reported. Our results are likely to be conservative because the model used travel distance rather than travel time; travel time may be high when a trip is taken on public transit, despite a short distance. The model assumes that people would travel to the nearest facility, but considerations such as preexisting health conditions, financial limitations, or gestational age may affect the choice of a facility. However, a national survey of patients undergoing abortion^33^ showed that 80% went to the nearest facility or to one that was within 15 miles of the nearest facility.

## Conclusions

In this cross-sectional study, greater travel distance to the nearest abortion care facility was associated with a lower abortion rate, suggesting that reducing travel distances to abortion facilities would increase access, even in states without restrictive laws. These results identify geographic areas with insufficient access to abortion care and could inform decisions about the location of new facilities. However, given the low population density of many counties with poor access, innovative strategies to meet the need for care are warranted.^34^ The Centers for Disease Control and Prevention included in its 10 essential public health services “assur[ing] an effective system that enables equitable access to the individual services and care needed to be healthy.”^35^ A public health approach to abortion would use strategies that have increased access to other forms of medical care, including expanding the types of clinicians who can offer care, mobile clinics, telemedicine, dispensing via lockboxes, and mail order delivery of medications.^36^ Such public health approaches will become even more important if the US Supreme Court gives states more leeway to regulate or ban abortion. New models of abortion care could rely less on facilities and increase access even for remote counties, leading to more equitable reproductive health care nationwide.

## Notes

Supplement. **eTable 1.** Reported Abortions, Women of Reproductive Age, Median Abortion Rate, Median Travel Distance, and Median County Proportion of Female Residents With Selected Sociodemographic Characteristics, by US State (2015) **eTable 2.** Estimated Increases in Abortions Under Travel Distance Scenarios, by Travel Distance to the Nearest Abortion Care Facility (n = 3107) **eTable 3.** Sensitivity Analysis: Decline in County-Level Abortion Rate in a Spatial Poisson Model, by Travel Time to the Nearest Abortion Care Facility (n = 1948) **eFigure 1.** Median Travel Distance in Miles to Closest Abortion Care Facility, by County (2015) **eFigure 2.** Estimated Abortion Rate per 1000 Female Residents of Reproductive Age in a Scenario With a Maximum Travel Distance of 30 Miles, by County of Residence (2015) **eFigure 3.** Estimated Abortion Rate per 1000 Female Residents of Reproductive Age in a Scenario With a Maximum Travel Distance of 5 Miles, by County of Residence (2015)
