# Supplementary material for: Association of Travel Distance to Nearest Abortion Facility With Rates of Abortion
Source: JAMA Netw Open. 2021 Jul 6;4(7):e2115530. doi: 10.1001/jamanetworkopen.2021.15530 (PMC8261612; doi:10.1001/jamanetworkopen.2021.15530)
Supplement: Supplement. — eTable 1. Reported Abortions, Women of Reproductive Age, Median Abortion Rate, Median Travel Distance, and Median County Proportion of Female Residents With Selected Sociodemographic Characteristics, by US State (2015) eTable 2. Estimated Increases in Abortions Under Travel Distance Scenarios, by Travel Distance to the Nearest Abortion Care Facility (n = 3107) eTable 3. Sensitivity Analysis: Decline in County-Level Abortion Rate in a Spatial Poisson Model, by Travel Time to the Nearest Abortion Care Facility (n = 1948) eFigure 1. Median Travel Distance in Miles to Closest Abortion Care Facility, by County (2015) eFigure 2. Estimated Abortion Rate per 1000 Female Residents of Reproductive Age in a Scenario With a Maximum Travel Distance of 30 Miles, by County of Residence (2015) eFigure 3. Estimated Abortion Rate per 1000 Female Residents of Reproductive Age in a Scenario With a Maximum Travel Distance of 5 Miles, by County of Residence (2015) [file jamanetwopen-e2115530-s001.pdf]

# Supplementary Online Content

Thompson KMJ, Sturrock HJW, Foster DG, Upadhyay UD. Association of travel distance to nearest abortion facility with rates of abortion. *JAMA Netw Open*. 2021;4(7):e2115530. doi:10.1001/jamanetworkopen.2021.15530

**eTable 1.** Reported Abortions, Women of Reproductive Age, Median Abortion Rate, Median Travel Distance, and Median County Proportion of Female Residents With Selected Sociodemographic Characteristics, by US State (2015)

**eTable 2.** Estimated Increases in Abortions Under Travel Distance Scenarios, by Travel Distance to the Nearest Abortion Care Facility (n = 3107)

**eTable 3.** Sensitivity Analysis: Decline in County-Level Abortion Rate in a Spatial Poisson Model, by Travel Time to the Nearest Abortion Care Facility (n = 1948)

**eFigure 1.** Median Travel Distance in Miles to Closest Abortion Care Facility, by County (2015)

**eFigure 2.** Estimated Abortion Rate per 1000 Female Residents of Reproductive Age in a Scenario With a Maximum Travel Distance of 30 Miles, by County of Residence (2015)

**eFigure 3.** Estimated Abortion Rate per 1000 Female Residents of Reproductive Age in a Scenario With a Maximum Travel Distance of 5 Miles, by County of Residence (2015)

This supplementary material has been provided by the authors to give readers additional information about their work.

© 2021 Thompson KMJ et al. *JAMA Network Open*.

**eTable 1. Reported Abortions, Women of Reproductive Age, Median Abortion Rate, Median Travel Distance, and Median County Proportion of Female Residents With Selected Demographic Characteristics, by US State (2015)[a]**

| State | No. of counties | No. of abortions | No. of female residents of reproductive age | Median abortion rate[b] | Median proportion ages 25 to 29 | Median proportion Black or other race/ ethnicity[c] | Median proportion married[d] | Median proportion with high school degree[e] | Median proportion foreign born[f] | Median proportion below federal poverty level[g] |
|---|---|---|---|---|---|---|---|---|---|---|
| Total | 1 948 | 428 720 | 37 276 590 | 9.9 | 15.5 | 4.6 | 51.5 | 27.0 | 2.6 | 21.3 |
| Alabama | 67 | 6 620 | 961 050 | 5.5 | 15.9 | 24.7 | 48.1 | 27.9 | 1.7 | 25.7 |
| Arizona | 15 | 12 640 | 1 296 680 | 11.6 | 16.5 | 8.0 | 49.6 | 26.5 | 7.4 | 26.2 |
| Colorado | 64 | 8 970 | 1 072 350 | 8.8 | 15.8 | 3.2 | 54.1 | 22.3 | 5.2 | 19.0 |
| Delaware | 3 | 2 890 | 179 990 | 17.3 | 17.0 | 28.8 | 46.3 | 24.8 | 6.7 | 17.0 |
| Georgia | 159 | 26 820 | 2 099 370 | 14.2 | 15.5 | 29.5 | 46.3 | 29.4 | 2.8 | 27.1 |
| Idaho | 44 | 1 690 | 311 320 | 5.5 | 15.2 | 3.0 | 57.4 | 30.4 | 4.1 | 21.4 |
| Illinois | 53 | 34 280 | 2 499 310 | 9.9 | 15.7 | 7.1 | 49.7 | 22.6 | 2.4 | 20.1 |
| Indiana | 92 | 9 550 | 1 291 070 | 6.0 | 15.1 | 2.2 | 52.7 | 29.0 | 1.8 | 18.7 |
| Kansas | 105 | 3 630 | 558 930 | 6.8 | 15.6 | 2.8 | 56.9 | 22.0 | 2.2 | 18.4 |
| Louisiana | 64 | 8 520 | 939 820 | 8.0 | 16.9 | 32.7 | 44.0 | 32.7 | 1.4 | 26.0 |
| Michigan | 83 | 26 280 | 1 892 790 | 10.7 | 14.9 | 3.8 | 52.8 | 26.6 | 1.9 | 23.1 |
| Minnesota | 87 | 9 230 | 1 049 650 | 8.6 | 15.1 | 3.5 | 55.9 | 22.7 | 2.3 | 16.5 |
| Mississippi | 82 | 4 700 | 601 390 | 6.9 | 16.1 | 38.6 | 43.4 | 27.6 | 0.9 | 31.0 |
| Missouri | 115 | 8 640 | 1 175 400 | 5.7 | 15.7 | 3.0 | 53.4 | 29.4 | 1.2 | 23.6 |
| Nebraska | 57 | 1 890 | 351 450 | 6.2 | 14.9 | 2.0 | 58.3 | 21.7 | 2.2 | 17.1 |
| New York | 62 | 88 760 | 4 048 540 | 17.9 | 15.3 | 7.0 | 47.0 | 23.8 | 4.1 | 19.0 |
| Ohio | 88 | 21 220 | 2 214 450 | 8.7 | 15.3 | 4.2 | 51.9 | 30.2 | 1.5 | 21.3 |
| Oregon | 36 | 7 850 | 770 670 | 9.7 | 16.1 | 5.7 | 51.3 | 27.0 | 5.2 | 24.6 |
| Pennsylvania | 67 | 32 030 | 2 418 790 | 10.6 | 15.3 | 4.3 | 50.8 | 31.8 | 1.7 | 18.1 |
| South Carolina | 46 | 11 030 | 937 780 | 12.1 | 15.9 | 35.1 | 44.2 | 28.8 | 2.4 | 27.2 |
| South Dakota | 18 | 580 | 124 330 | 4.1 | 16.3 | 5.8 | 51.5 | 21.9 | 1.9 | 16.5 |

© 2021 Thompson KMJ et al. *JAMA Network Open*.

| State | No. of counties | No. of abortions | No. of female residents of reproductive age | Median abortion rate[b] | Median proportion ages 25 to 29 | Median proportion Black or other race/ ethnicity[c] | Median proportion married[d] | Median proportion with high school degree[e] | Median proportion foreign born[f] | Median proportion below federal poverty level[g] |
|---|---|---|---|---|---|---|---|---|---|---|
| Texas | 254 | 54 200 | 5 577 580 | 10.0 | 15.6 | 5.4 | 52.1 | 28.4 | 6.8 | 21.1 |
| Utah | 29 | 3 120 | 631 170 | 3.7 | 14.7 | 3.5 | 58.8 | 26.4 | 3.9 | 16.0 |
| Vermont | 14 | 1 120 | 116 670 | 9.5 | 15.0 | 3.3 | 49.8 | 28.1 | 3.8 | 17.4 |
| Virginia | 133 | 18 500 | 1 677 520 | 8.6 | 15.6 | 16.0 | 50.0 | 27.4 | 3.0 | 18.2 |
| Washington | 39 | 17 230 | 1 390 380 | 12.2 | 16.2 | 7.3 | 51.5 | 26.1 | 7.0 | 21.7 |
| Wisconsin | 72 | 6 720 | 1 088 170 | 5.2 | 14.9 | 3.1 | 55.2 | 26.6 | 2.2 | 16.7 |

[a] Columns may not sum to total due to rounding.

[b] Rates are population-weighted median per 1000 female residents aged 15 to 44.

[c] Includes Asian, Native American, Alaska Native, Native Hawaiian, Pacific Islander, and multiracial residents. Denominator is female residents aged 15 to 44.

[d] Denominator is female residents over age 18.

[e] HS = High school or equivalent degree. Denominator is female residents aged 18 to 44.

[f] Denominator is all female residents.

[g] Denominator is households with female residents aged 15 to 44.

© 2021 Thompson KMJ et al. *JAMA Network Open*.

**eTable 2. Estimated Increases in Abortions Under Travel Distance Scenarios, by Travel Distance to the Nearest Abortion Care Facility (n = 3107)**

| Travel distance, miles[a] | No. of counties | No. of female residents of reproductive age[b] | Actual travel distance | Travel distance scenario <30 m | | Travel distance scenario <5 m | |
|---|---|---|---|---|---|---|---|
| | | | No. of abortions | No. of abortions | No. of additional abortions | No. of abortions | No. of additional abortions |
| Total | 3 107 | 62 539 010 | 696 470 | 714 660 | 18 190 | 767 390 | 70 920 |
| <5 | 107 | 16 903 950 | 289 790 | 289 790 | NA | 289 790 | NA |
| 5 to <15 | 261 | 22 987 240 | 270 550 | 270 550 | NA | 285 360 | 14 810 |
| 15 to <30 | 282 | 6 922 720 | 52 980 | 52 980 | NA | 66 000 | 13 020 |
| 30 to <60 | 806 | 7 208 400 | 41 800 | 47 320 | 5 510 | 58 940 | 17 140 |
| 60 to <120 | 1 089 | 6 705 880 | 34 520 | 42 690 | 8 170 | 53 180 | 18 660 |
| ≥120 | 562 | 1 810 820 | 6 830 | 11 330 | 4 510 | 14 120 | 7 290 |

Abbreviation: NA, not applicable.

[a] To convert to kilometers, multiply by 1.6.

[b] Includes women and girls aged 15 to 44 years.

© 2021 Thompson KMJ et al. *JAMA Network Open*.

**eTable 3. Sensitivity Analysis: Decline in County-Level Abortion Rate in a Spatial Poisson Model, by Travel Time to the Nearest Abortion Care Facility (n = 1948)**

| Travel time, minutes | No. of counties | No, of female residents of reproductive age[a] | No. of abortions | Median abortion rate[b] | Range | Coefficient (95% CI) | |
|---|---|---|---|---|---|---|---|
| | | | | | | Unadjusted | Adjusted[c] |
| <5 | 5 | 1 248 140 | 40 270 | 28.3 | 25.1 to 63.6 | 1 [Reference] | 1 [Reference] |
| 5 to <15 | 133 | 14 650 660 | 229 090 | 14.2 | 1.0 to 35.0 | 0.58 (0.52 to 0.64) | -0.41 (-0.50 to -0.32) |
| 15 to <30 | 117 | 7 199 940 | 74 310 | 10.0 | 0.7 to 19.6 | 0.24 (0.18 to 0.30) | -0.43 (-0.52 to -0.33) |
| 30 to <60 | 371 | 5 937 640 | 40 950 | 6.7 | 0.2 to 22.9 | -0.05 (-0.11 to 0.01) | -0.64 (-0.74 to -0.54) |
| 60 to <120 | 779 | 5 634 540 | 32 130 | 5.3 | 0 to 19.3 | -0.10 (-0.17 to -0.04) | -0.75 (-0.85 to -0.65) |
| ≥120 | 543 | 2 605 680 | 11 970 | 4.3 | 0 to13.0 | -0.13 (-0.20 to -0.05) | -0.76 (-0.87 to -0.65) |

[a] Includes women and girls aged 15 to 44 years.

[b] Indicates population-weighted travel time category median per 1000 women aged 15 to 44 years.

[c] Adjusted for county-level proportion of female residents aged 25 to 29, Black or other race/ethnicity, married, high school degree or equivalent, foreign-born residents, households below the federal poverty level, and state abortion policies.

© 2021 Thompson KMJ et al. *JAMA Network Open*.

**eFigure 1. Median Travel Distance in Miles to Closest Abortion Care Facility, by County (2015)**

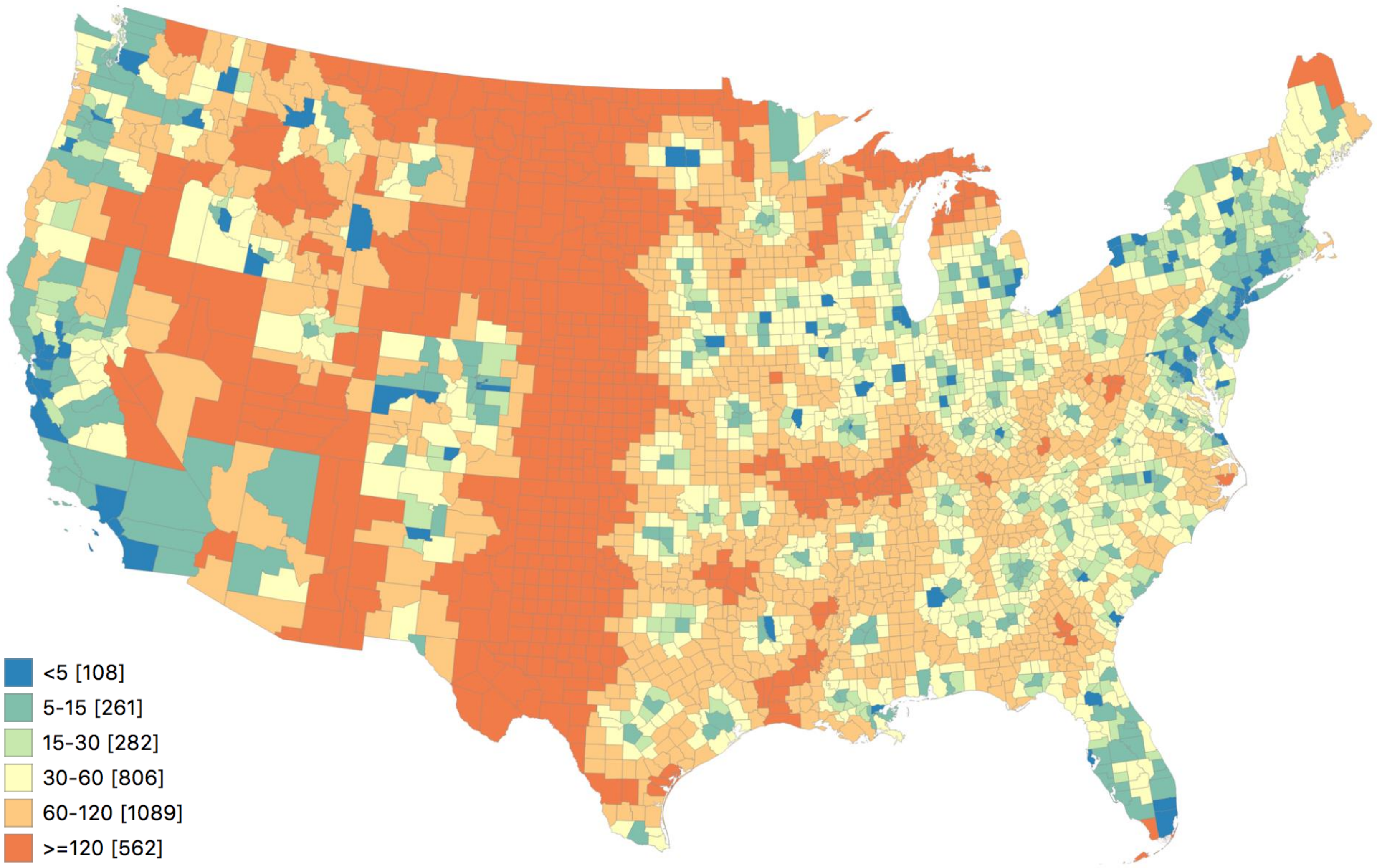


Note: Conversion factor from miles to kilometers is 1.6.

© 2021 Thompson KMJ et al. *JAMA Network Open*.

**eFigure 2. Estimated Abortion Rate per 1000 Female Residents of Reproductive Age in a Scenario With a Maximum Travel Distance of 30 Miles, by County of Residence (2015)**

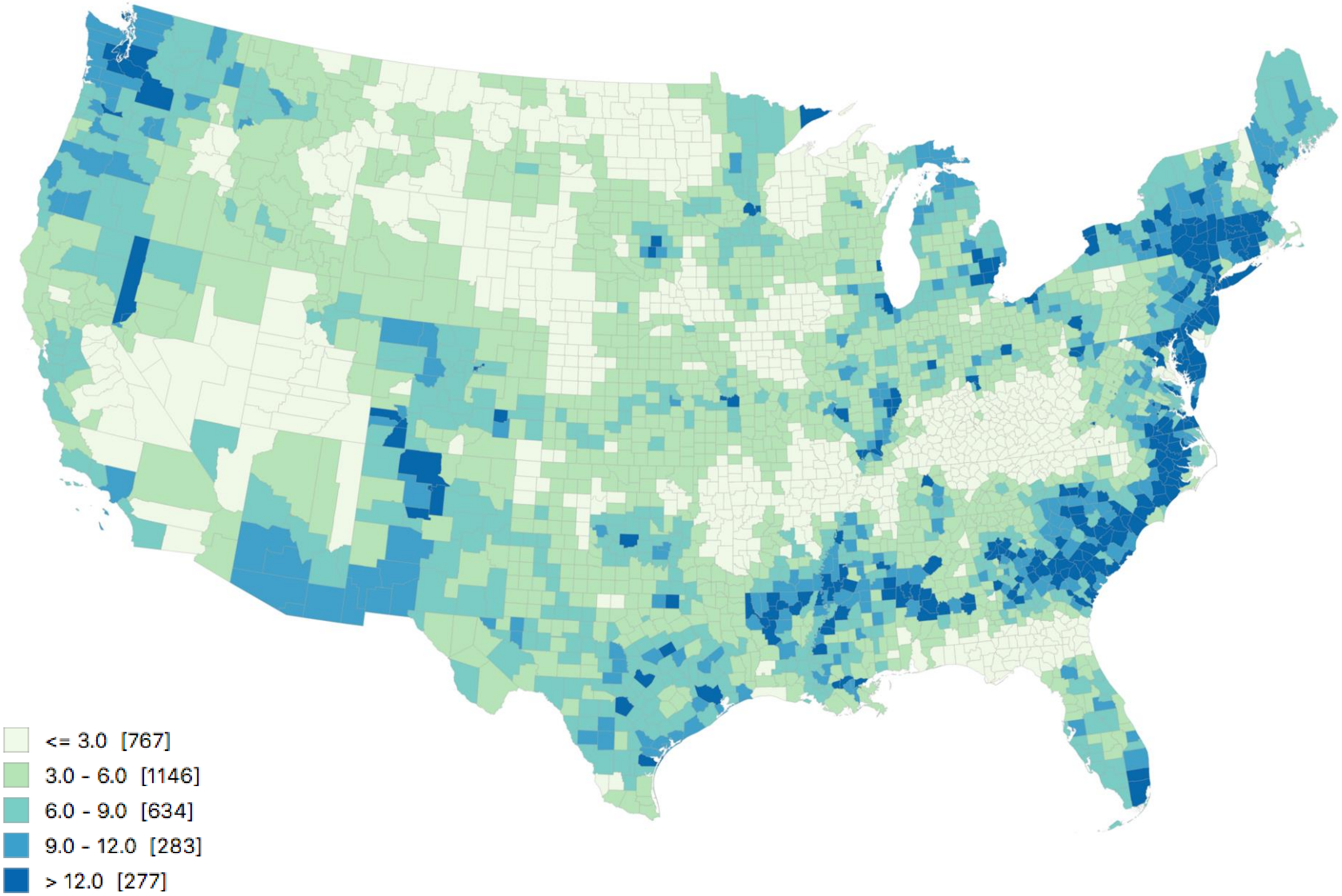


© 2021 Thompson KMJ et al. *JAMA Network Open*.

**eFigure 3. Estimated Abortion Rate per 1000 Female Residents of Reproductive Age in a Scenario With a Maximum Travel Distance of 5 Miles, by County of Residence (2015)**

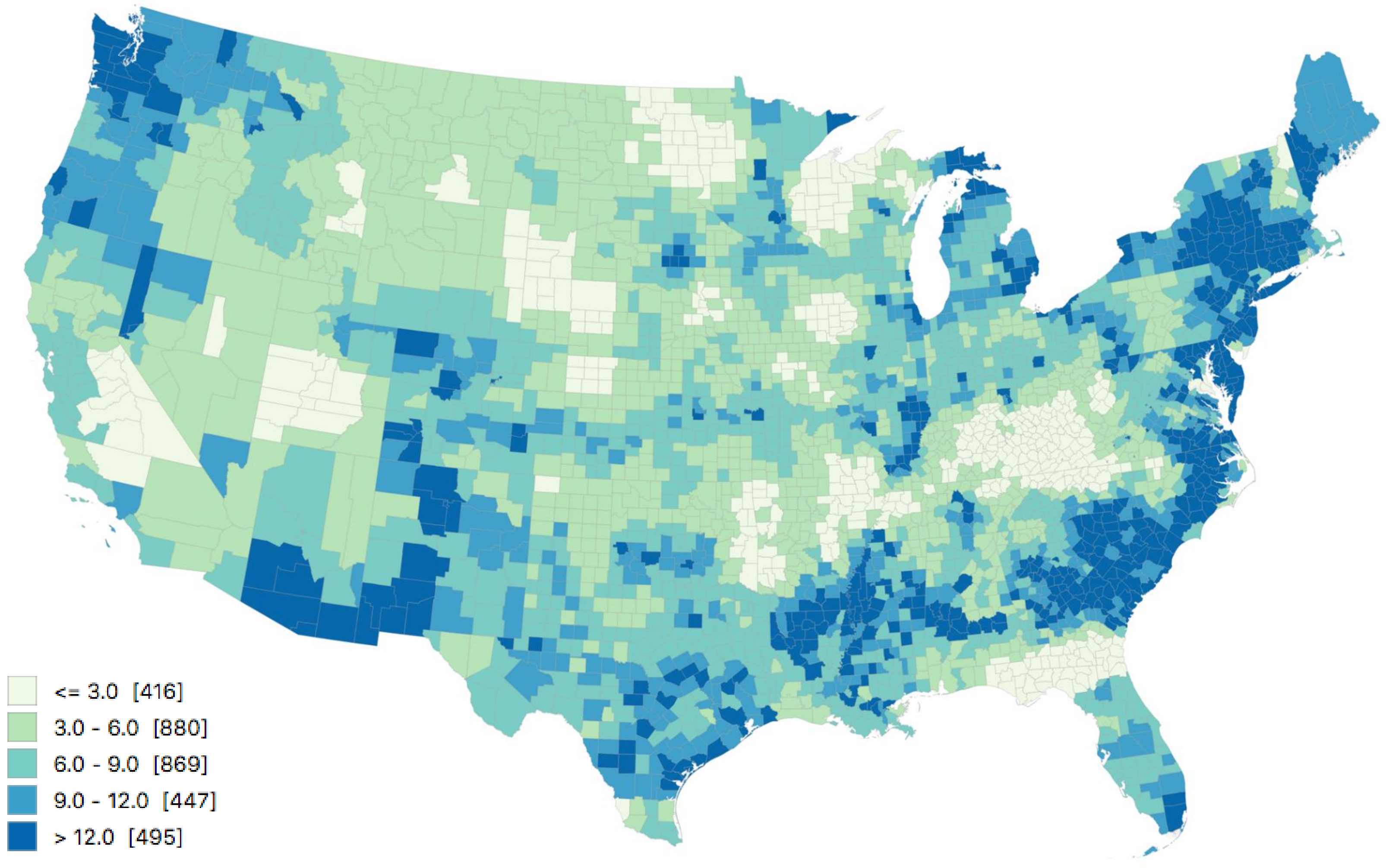


© 2021 Thompson KMJ et al. *JAMA Network Open*.
